# Potenzial grüner Wasserstoff: langer Weg der Entwicklung, kurze Zeit bis zur Umsetzung

**DOI:** 10.1007/s10273-021-2821-9

**Published:** 2021-01-19

**Authors:** Mirko Kruse, Jan Wedemeier

**Affiliations:** Hamburgischen WeltWirtschaftsInstitut gemeinnützige GmbH (HWWI), Fahrenheitstr. 1, 28359 Bremen, Deutschland

**Keywords:** R10, R11, Q2

## Abstract

Das Thema Wasserstoff wird seit längerem diskutiert, doch jüngst hat es im Zuge des Corona-Konjunkturpakets des Bundes einen spürbaren Bedeutungszuwachs erfahren. Eine Wasserstoffwirtschaft verspricht die notwendige nachhaltige Transformation der Wirtschaft bei gleichzeitigem Erhalt der Industriestruktur, zusätzliche Arbeitsplätze und Wachstumspotenziale in einer resilienteren Ökonomie.

## Abstract (en)

The aim of the paper is to examine the areas in which there is a potential for hydrogen use in Germany. The race to the top has already started. Green hydrogen’s potential is immense. The long road to development has already been undertaken, and now a rapid implementation is required to prepare this technological and structural transformation for international competition. The paper considers from an administrative perspective how the production and application potentials are distributed in Germany, from which recommendations for further actions are derived.

Um die Erderwärmung auf unter 2°C zu begrenzen, hat sich Deutschland, in Einklang mit den Zielen der Klimakonferenz von Paris, zu einem ambitionierten $CO_2$-Reduktionspfad verpflichtet. So sollen bis 2050 die Treibhausgasemissionen in $CO_2$-Äquivalenten gegenüber 1990 um 80 % bis 95 % gesenkt werden. Zu diesem Zweck ist für alle Wirtschaftssektoren (mit Ausnahme der Landwirtschaft) bis Mitte des Jahrhunderts weitgehende Treibhausgasneutralität zu erreichen (IPCC, 2020; BMU, 2020; Hebling et al., 2019). Die bisher geplanten und in Umsetzung befindlichen Maßnahmen zur Emissionsreduktion werden jedoch bis 2050 voraussichtlich lediglich 61 % der $CO_2$-Emissionen gegenüber 1990 einsparen, sodass eine deutliche Intensivierung der Maßnahmen vonnöten ist (Gerbert et al., 2018; Lebling et al., 2020).

Teil einer nachhaltigen Transformation der Wirtschaft ist eine umfassende Energiewende, die den Anteil fossiler, nicht erneuerbarer Energieträger auf ein Minimum reduziert. Da erneuerbare Energieträger jedoch Eigenschaften aufweisen, die eine weniger flexible Anwendung erlauben, unter anderem die Wetterabhängigkeit der Erzeugung oder Erschwernisse bei Transport und Lagerung, sind Anpassungen notwendig, um die Wirtschaft auf eine erneuerbare Basis zu stellen. In diesem Zusammenhang bietet Wasserstoff die Möglichkeit, als „Bindeglied zwischen der elektrischen und der stofflichen Welt“ (Hebling et al., 2019: 11) zu fungieren. Indem erneuerbarer Strom zur Erzeugung von Wasserstoff genutzt wird, der später wieder in Energie umgewandelt werden kann, würden die spezifischen Nachteile erneuerbarer Energien umgangen. Dieses Verfahren wird auch unter „Power-to-Gas (P2G)“ zusammengefasst.

**Mirko Kruse** ist Junior Researcher am Hamburgischen WeltWirtschaftsInstitut (HWWI).

**Dr. Jan Wedemeier** ist dort Senior Researcher und Leiter des Forschungsbereichs „Ökonomie der Städte und Regionen“.

Zwar wird die Wasserstofftechnologie schon seit längerem diskutiert, das Thema hat jedoch im Zuge des Corona-Konjunkturpakets des Bundes einen spürbaren Bedeutungszuwachs erfahren. So wurde 2020 von der Bundesregierung eine Nationale Wasserstoffstrategie vorgelegt und gleichzeitig mit der Ankündigung verknüpft, finanzielle Mittel zur Überwindung der Corona-Krise zum Aufbau einer Wasserstoffwirtschaft zu verwenden (BMF, 2020; Bach et al., 2020). Ähnliche Erwägungen leiten auch die Europäische Kommission, die Wasserstoff als wichtige Säule für den geplanten European Green Deal betrachtet (Europäische Kommission, 2020b). Eine Wasserstoffwirtschaft verspricht, die notwendige nachhaltige Transformation der Wirtschaft bei gleichzeitigem Erhalt der Industriestruktur zu erreichen und zusätzliche Arbeitsplätze, Wachstumspotenziale sowie eine resilientere Ökonomie zu schaffen. Allein in Nordrhein-Westfalen könnte durch Wasserstoff rund ein Viertel der jährlichen $CO_2$-Emissionen eingespart werden (MWIDE, 2020). Es ist zu erwarten, dass nach weitgehender Überwindung der Folgen der Corona-Pandemie der Themenbereich Nachhaltigkeit wieder stärker in den öffentlichen Fokus rückt und Wasserstoff eine zentrale Rolle in der Diskussion einnehmen wird.

Wasserstoff kann mithilfe unterschiedlicher Verfahren erzeugt werden, wobei das größte Potenzial im Elektrolyseprozess gesehen wird, bei dem Wasser und elektrische

Energie zu Wasserstoff verbunden werden (Hebling et al., 2019). Einen Beitrag zur Dekarbonisierung leistet Wasserstoff dann, wenn er mithilfe erneuerbarer Energie erzeugt wird (grüner Wasserstoff). Dem stehen weitere Varianten gegenüber, die sich hinsichtlich der Erzeugung unterscheiden, beispielsweise „grauer“ Wasserstoff auf Basis von Energie aus Erdgas oder „blauer“ Wasserstoff aus fossilen Energieträgern mit anschließender Abscheidung und Lagerung des entstehenden $CO_2$ (Kumar, 2015; IN4Climate.NRW, 2019).

## Wieso über Wasserstoff diskutiert wird

Seit 1975 hat die weltweite Nachfrage nach Wasserstoff sich bereits mehr als verdreifacht, was die vielfältigen Nutzungsmöglichkeiten unterstreicht (Weichenhain et al., 2020). Der zentrale Vorteil von Wasserstoff besteht darin, die Nachteile erneuerbarer Energie gegenüber fossilen Energieträgern auszugleichen. So kann in manchen Bereichen in Akkus gespeicherte Energie eine mögliche Alternative zu fossilen Energieträgern darstellen (z. B. in Fahrzeugen mit Elektroantrieben im Innenstadtbereich und auf Kurzstrecken), allerdings reicht die Energiedichte von Batterien in anderen Bereichen nicht aus (z. B. Fahrzeuge mit Elektroantrieben auf Langstrecken).

An dieser Stelle kommt Wasserstoff als mögliche Ergänzung ins Spiel, der eine höhere Energiedichte aufweist und daher auch in industriellen Bereichen Anwendung finden kann. Wasserstoff lässt sich, ähnlich wie Flüssiggas, lagern oder mithilfe von Pipelines oder Schiffen auch über größere Entfernungen transportieren. Zudem entsteht bei der Verbrennung von Wasserstoff lediglich wieder Wasser als Reststoff. Entsprechend verbindet sich mit der Nutzung von Wasserstoff ebenso die Hoffnung auf saubere Luft in Industriegebieten und an Verkehrswegen (IEA, 2019).

Die Versprechen einer Wasserstoffwirtschaft lassen bereits erahnen, dass der Bedarf an Wasserstoff und an erneuerbarer Energie zur Wasserstofferzeugung immens sein wird. Hinzu kommt, dass die Erzeugungskosten von Wasserstoff hoch sind, der Prozess also mit einem signifikanten Energieverlust verbunden ist. Je nach Elektrolyseverfahren beträgt der Wirkungsgrad zwischen 61 % bis 68 % (AEL) und 77 % bis 81 % (SOEC) (MWIDE, 2020).[1] Zwar wird erwartet, dass bis 2030 durch Skaleneffekte und sinkende Energiepreise eine Kostenreduktion von 30 % erzielt werden kann, nichtsdestotrotz decken die aktuellen Erzeugungskapazitäten für erneuerbare Energie den zukünftigen Bedarf lediglich in Ansätzen (IEA, 2019). Im Jahr 2018 lag der Anteil erneuerbarer Energien im deutschen Strommix bei etwa 38 %, wobei Bereiche wie Wärmeerzeugung oder Mobilität hierbei noch nicht berücksichtigt wurden (Hebling et al., 2019). Ein deutlicher Ausbau erneuerbarer Energien wird die Planungen einer Wasserstoffwirtschaft zwangsläufig flankieren müssen. Der Schwerpunkt wird hier voraussichtlich auf Windenergie (On- und Offshore) liegen (Joest et al., 2009; Europäische Kommission, 2020a).

1 Elektrolysetechnologien: Alkalisch (AEL) und Hochtemperatur (SOEC).

## Ausgangslage und Entwicklungsprognosen

Seitdem die Bundesregierung kürzlich die erste Nationale Wasserstoffstrategie vorgelegt hat, positionieren sich zunehmend auch Bundesländer wie Nordrhein-Westfalen, Bayern oder die norddeutschen Bundesländer mit eigenen Strategien zu diesem Thema (BMWi, 2020; MWIDE, 2020; STMWI, 2020; IHK Nord, 2019). Neben Deutschland arbeiten auch Australien, Japan, Südkorea und die Niederlande an eigenen Wasserstoffstrategien (IN4Climate.NRW, 2019).

Bereits heute werden rund 55 TWh bis 60 TWh Wasserstoff in Deutschland produziert und verbraucht, allerdings handelt es sich hierbei überwiegend um grauen Wasserstoff aus Erdgas und lediglich zu etwa 5 % um grünen Wasserstoff (DLR et al., 2015; Merten et al., 2020). Für den Zeitraum bis 2030 ist geplant, die Erzeugungskapazität von grünem Wasserstoff aus erneuerbaren Energien in Deutschland im Stromnetz auf 1 GW bis 5 GW zu steigern (respektive zwischen 50 GW und 80 GW bis 2050). Dies entspräche 2030 einer Wasserstoffproduktion von bis zu 14 TWh und würde nicht nur deutlich mehr erneuerbare Energie erfordern, sondern auch ein hohes Ausbautempo und eine deutliche technologische Weiterentwicklung der Erzeugungskapazitäten voraussetzen (BMWi, 2020). Dem steht bis 2030 eine erwartete Nachfrage von 4 TWh bis 20 TWh Wasserstoff in Deutschland gegenüber, zuzüglich 6 TWh in der Stahlerzeugung. Bis 2050 kann dieser Bedarf auf 250 TWh bis 800 TWh (zuzüglich 38 TWh bis 56 TWh zur Stahlerzeugung) ansteigen, wenn sowohl in verschiedenen Industriebereichen als auch im Verkehrsbereich auf Wasserstoff umgerüstet wird (Hebling et al., 2019).

Der Umfang des Vorhabens einer Wasserstoffwirtschaft legt nahe, auch die Perspektive als europäisches Gemeinschaftsprojekt zu berücksichtigen. So sieht die europäische Wasserstoffstrategie der Europäischen Kommission vor, bis 2024 1 Mio. t und bis 2030 10 Mio. t grünen Wasserstoff zu erzeugen. Die Elektrolysekapazität soll dabei europaweit von 6 GW auf 40 GW ansteigen, sodass bis zum Jahr 2030 ein europäischer Wasserstoffmarkt etabliert wäre. Es ergibt sich also ein Zeitfenster von zehn Jahren für den Markthochlauf der Wasserstofftechnologien im europäischen Kontext. Erwartet wird bis 2050 ein Investitionsvolumen von 488 Mrd. Euro (Europäische Kommission, 2020b).

## Wo der Wasserstoff erzeugt wird

Bis 2050 wird ein Strommix erwartet, der bei erneuerbaren Energien zu 20 % von Photovoltaik, zu 38 % von Offshore-Wind und zu 42 % von Onshore-Wind gedeckt wird (Weichenhain et al., 2020). Elektrolyseure zur Wasserstoffproduktion werden dabei voraussichtlich besonders in solchen Regionen eingesetzt, in denen die Kosten der Stromerzeugung bei unter 3 ct/kWh liegen und wo möglichst durchgängig erneuerbare Energie erzeugt werden kann (Hebling et al., 2019). Auf nationaler Ebene werden hier besonders die norddeutschen Bundesländer aufgrund ihrer geografischen Nähe zu den Offshore-Windparks in Nord- und Ostsee eine wichtige Rolle bei der Wasserstoffproduktion spielen. Hinzu kommen hohe Erzeugungspotenziale für Onshore-Windenergie vorwiegend in ebendiesen norddeutschen Bundesländern Niedersachsen, Schleswig-Holstein, Mecklenburg-Vorpommern, wie auch Brandenburg und Sachsen-Anhalt. Hinsichtlich Energie aus Photovoltaik werden die größten Potenziale für Freiflächenanlagen überwiegend in Süd- sowie in Nordostdeutschland gesehen, vorwiegend in den Bundesländern Bayern, Baden-Württemberg und Brandenburg (Merten et al., 2020).

Angesichts des hohen prognostizierten Wasserstoffbedarfs und einer erst aufzubauenden Erzeugungsinfrastruktur mit begrenzter Kapazität wird auch über die Möglichkeit eines Imports von grünem Wasserstoff spekuliert (BMWi, 2020). Zum einen legen Berechnungen nahe, dass die Kosten für Kühlung und Transport eine lokale Erzeugung von Wasserstoff aus erneuerbaren Energien deutlich wirtschaftlicher erscheinen lassen (Merten et al., 2020). Andererseits arbeitet Deutschland bereits heute unter dem Titel „H2 Global“ am Aufbau von Wasserstoffpartnerschaften mit dem Ausland, um Wasserstoffimporte aus sonnen- und windreichen Regionen sicherzustellen (Stratmann, 2020). Erwartet wird, bis 2050 etwa drei Viertel der jährlichen Wasserstoffnachfrage durch Importe zu bedienen (MWIDE, 2020).

Erneut kommt den norddeutschen Bundesländern in diesem Zusammenhang eine potenziell wichtige Rolle zu, da diese mit ihren Seehäfen Einspeisepunkte ersten Grades für importierten Wasserstoff wären. Hinzu kommen geologische Faktoren wie das Vorhandensein von unterirdischen Salzkavernen, in die Wasserstoff eingeleitet und gespeichert werden könnte und die vorwiegend in Norddeutschland existieren (MWIDE, 2020; BMWi, 2011).

Mögliche Anwendungsfelder für Wasserstoff werden in nahezu allen energieintensiven Industrien (unter anderem Glasindustrie, Stahl, chemische Industrie) gesehen, wo bereits kurzfristig eine Einkopplung von Wasserstoff in bestehende Prozesse möglich ist. Diese Industrien liefern vor allem Grund- und Werkstoffe für nachstehende Industrien wie Fahrzeugbau, Verpackungen oder auch Bauwirtschaft. Daneben ist der Verkehrsbereich zu benennen. Kurz- und mittelfristig wird im Gebäudesektor kein relevanter Wasserstoffbedarf erwartet, allerdings könnte sich dies bei veränderten Rahmenbedingungen ändern. Denkbar wäre hier eine dezentrale Strom- und Wärmeerzeugung auf Basis von Wasserstoff, was jedoch ein getrenntes Wasserstoffnetz erfordern würde, sowie eine Beimischung von Wasserstoff zur Wärmeerzeugung ins vorhandene Erdgasnetz. Möglich wäre auch eine Rückverstromung des Wasserstoffs. Diese Variante wird allerdings, angesichts des hohen Energieaufwands der Wasserstofferzeugung, nicht als effizient erachtet (Hebling et al., 2019; Hydrogel Council, 2017). Auch weitere Branchen wie die Luftfahrtindustrie werden voraussichtlich auf synthetische Kraftstoffe wie grünen Wasserstoff angewiesen sein, um das Ziel der Klimaneutralität erreichen zu können (Stratmann, 2020).

Die hohe Zahl von potenziellen Anwendern steht dabei der begrenzten Verfügbarkeit und den hohen Erzeugungskosten von Wasserstoff entgegen. Besonders in der Entwicklungsphase einer Wasserstoffwirtschaft wird also eine Priorisierung solcher Sektoren, die ein besonders hohes Emissionsreduktionspotenzial aufweisen, notwendig sein. Nachfolgend sollen daher die beiden Bereiche näher betrachtet werden, denen die günstigsten Aussichten für Wasserstoff prognostiziert werden.

## Anwendungspotenziale in der Industrie

Der industrielle Sektor in Deutschland ist, nach der Energiewirtschaft, mit 23 % der Kohlendioxidemissionen in Deutschland (Stand 2018) der größte Emittent von Treibhausgasen, was entsprechend hohe Reduktionspotenziale begründet (BMU, 2020). Da Technologien zur Abscheidung und Speicherung von $CO_2$ (CCS – Carbon Capture and Storage) oder zur Nutzung (CCU – Carbon Capture and Utilization) unter Akzeptanzvorbehalt stehen und hinsichtlich möglicher Folgewirkungen nicht abschließend bewertet werden können, kommt der Nutzung von Wasserstoff eine umso höhere Bedeutung zu, um Emissionen im Produktionsprozess grundsätzlich zu vermeiden (Hebling et al., 2019). Dabei wird der Handlungsdruck absehbar weiter zunehmen, wenn die Marktpreise für $CO_2$-Zertifikate steigen.

Ein Beispiel für Wasserstoff in emissionsintensiven Industrien wäre die Stahlerzeugung, die in Deutschland jährlich rund 7 % der $CO_2$-Emissionen verursacht. Bisher wird die Reduktion von Eisenerz mit Hilfe von Kokskohle durchgeführt, da im Herstellungsprozess besonders hohe Temperaturen benötigt werden.[2] Aktuell laufen erste Tests in Hochöfen, die einen Teil von Koks durch Wasserstoff substituieren. Entsprechende Mengen an Wasserstoff und vollständig umgerüstete Hochöfen vorausgesetzt könnte auf diese Weise perspektivisch Stahl nahezu treibhausgasneutral produziert werden (Weichenhain et al., 2020). Ein weiteres industrielles Anwendungsfeld für Wasserstoff findet sich in der Chemieindustrie, wo Wasserstoff als Rohstoff beispielsweise zur Herstellung von Ammoniak oder Methanol benötigt wird. Aktuell wird hier grauer Wasserstoff verwendet, der ohne Anpassungen der Produktionsanlagen durch grünen Wasserstoff ersetzt werden kann. Weitere Substituierungspotenziale ergeben sich im Raffineriebereich, wo (grauer) Wasserstoff aktuell zur Entschwefelung von Kraftstoffen verwendet wird, sowie in der Glasschmelze, die ebenfalls vorwiegend auf Erdgas beruht (Hebling et al., 2019).

Abbildung 1 zeigt, wie sich die genannten Industriebereiche geografisch in Deutschland auf Kreisebene verteilen. Hierzu wurde der Mittelwert des Beschäftigungsanteils der vier Wirtschaftszweige im Verarbeitenden Gewerbe gebildet, die als besonders aussichtsreich für Wasserstoffanwendungen identifiziert wurden. Mit einem Anteil von 11,09 % an der Gesamtbeschäftigung ist im fränkischen Kronach die höchste Konzentration in den vier Industriesektoren festzustellen, während der Mittelwert aller Kreise und kreisfreien Städte bei 0,64 % liegt. Es zeigt sich also, dass die durchschnittliche Wirtschaftsstruktur für viele Regionen des Landes keine herausragende Relevanz für Wasserstoffanwendungen begründet, während es gleichzeitig einige Kreise gibt, die eine besondere Betroffenheit aufweisen werden. Hierzu zählt besonders Oberbayern, wo neben Kronach auch die Regionen mit den zweit- und dritthöchsten Werten zu finden sind: Altönning (9,67 %) und Wunsiedel im Fichtelgebirge (5,89 %), ehe mit Holzminden (5,18 %) eine Region im südlichen Niedersachsen folgt. Weitere Schwerpunkte finden sich in Nordrhein-Westfalen, im Saarland sowie in Sachsen-Anhalt.

## Anwendungspotenziale im Verkehr

Mit einem Anteil von rund 21 % der deutschlandweiten $CO_2$-Emissionen (Stand 2018) liegt der Verkehrsbereich nur geringfügig hinter der Industrie (BMU, 2020). Es ist gleichzeitig zu erwarten, dass der gesellschaftliche Mobilitätsbedarf eher zu- als abnehmen wird, sodass reine Veränderungen des Nutzungsverhaltens (z. B. Sharing-Modelle) nicht ausreichen und Anpassungen der Antriebssysteme unumgänglich sein werden (Kruse und Wedemeier, 2020; Bludau et al., 2020).

2 Im Reduktionsprozess wiederum entsteht unter anderem Wasserstoff als Reduktionsgas.

Abbildung 1
**Vergleich potenzieller Wasserstoffnutzung im Verarbeitenden Gewerbe, 2018**

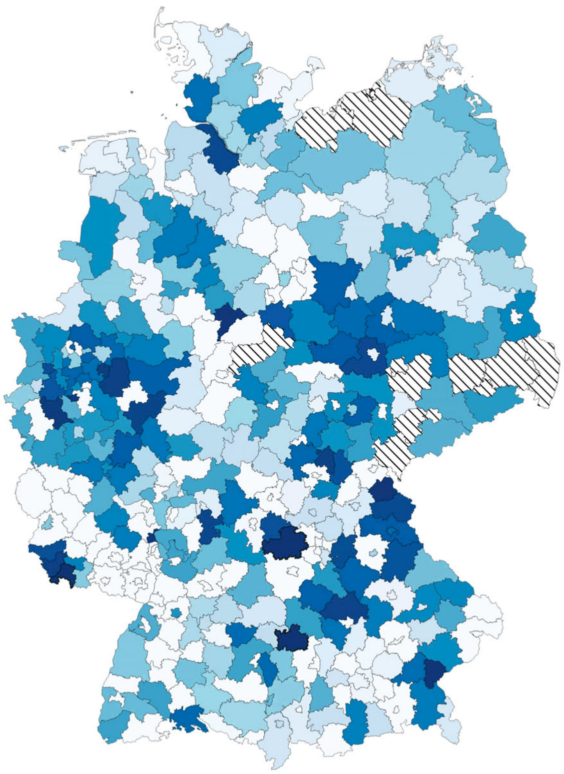

Hinweis: Die Farbskala ist nach Intensität abgestuft, wobei jedem Kreis eine eigene Farbgebung zukommt; schraffiert: keine Daten.

Anmerkung: Mittelwert des Beschäftigungsanteils von vier für Wasserstoffanwendungen besonders aussichtsreiche Wirtschaftszweige: WZ08-19: Kokerei und Mineralölverarbeitung; WZ08-20: Herstellung von chemischen Erzeugnissen; WZ08-23: Herstellung von Glas und Glaswaren, Keramik, Verarbeitung von Steinen und Erden; WZ08-24: Metallerzeugung und -bearbeitung (Statistisches Bundesamt, 2008).

Quelle: Statistisches Bundesamt (2019); eigene Darstellung.

Die Entscheidung, welche Antriebstechnologien für Fahrzeuge sich langfristig durchsetzen werden, ist indes noch nicht getroffen. Nichtsdestotrotz darf davon ausgegangen werden, dass Wasserstoff mit einer Brennstoffzelle in schweren Fahrzeugen mit hohen Reichweiten wie Schienenverkehr, Bussen, Lastkraftwagen (Lkw), Binnenschiffen oder Zugmaschinen sowie in Nutzfahrzeugen eingesetzt werden wird, da hier Vorteile gegenüber Batterieantrieben zu sehen sind (Hebling et al., 2019; Hydrogen Council, 2017). Diese Vorteile umfassen beispielsweise die bessere Speicherfähigkeit von Wasserstoff im Vergleich zu elektrischer Energie, eine schnellere Betankung von Fahrzeugen sowie eine höhere gravimetrische Energiedichte (Energie/Gewicht), die besonders im Schwerlast- und Nutzverkehr benötigt wird. Entsprechende Praxisversuche laufen bereits in unterschiedlichen Verkehrsmitteln. Zwar ist die Zahl der Lkw und Sattelzugmaschinen (zwischen 18 t und 44 t) in Deutschland mit rund 230.000 Fahrzeugen deutlich geringer als die Zahl von Personenkraftwagen (Pkw), jedoch entfallen auf jene Fahrzeuge die Hälfte der Emissionen des Straßengüterverkehrs (Wietschel et al., 2015). Der Wasserstoffbedarf für Lkw in Deutschland bis 2030 wird mit 0 TWh bis 2 TWh angegeben, wobei bis 2050 ein deutlicher Anstieg auf 130 TWh bis 160 TWh erwartet wird (Hebling et al., 2019).

Auch im Pkw-Bereich besteht ein erhebliches Potenzial für Brennstoffzellenantriebe. Gerade vor dem Hintergrund, dass Brennstoffzellenantriebe, anders als Elektromotoren, auf einer weitgehend ähnlichen Funktionsweise wie klassische Verbrenner basieren, ist die Wasserstofftechnologie für die deutsche Automobilindustrie von Interesse. Durch eine großflächige Nutzung von Wasserstoff könnten theoretisch bis zu 80 % der $CO_2$-Emissionen des Verkehrssektors eingespart werden (Joest et al., 2009). Angesichts der begrenzten Verfügbarkeit von Wasserstoff wird jedoch der Fokus, zumindest in der Hochlaufphase, auf Lkw und größeren Nutzfahrzeugen liegen, die eine höhere $CO_2$-Vermeidung ermöglichen als der Individualverkehr.

Abbildung 2 zeigt, wie sich der Anteil von Lkw und Sattelzugmaschinen am gesamten Fahrzeugbestand auf Kreisebene in Deutschland verteilt. Hierzu wurde der Mittelwert von Lkw - und Sattelzugmaschinenanteil gebildet, wobei unterstellt wird, dass jene Fahrzeuge zwischen 18 t und 44 t besondere Nutzungspotenziale für Wasserstoff aufweisen und zeitnah umgerüstet werden können.[3]

Während der Mittelwert des Anteils von Lkw und Sattelzugmaschinen am Fahrzeugbestand in allen Kreisen und kreisfreien Städten bei 4,94 % liegt, ragen einzelne Regionen heraus: So ist mit 9,53 % der Anteil im Landkreis Cham besonders hoch, gefolgt vom Landkreis Rottal-Inn (9,42 %) und dem Landkreis Freyung-Grafenau (9,42 %), welche alle in Bayern liegen. Niedrige Werte weisen gleichzeitig Wolfsburg (1,57 %), Ingolstadt (2,12 %) und Oberhausen (2,31 %) auf. Es wird deutlich, dass die Verteilung von Fahrzeugen, die Potenziale für Wasserstoffanwendungen aufweisen, ausgeglichener ist als die Industrieverteilung (vgl. Abbildung 1). Allerdings bilden sich auch hier regionale Schwerpunkte, besonders in Südostdeutschland und in den norddeutschen Bundesländern, was die hohe Bedeutung des Logistiksektors in diesen Regionen unterstreicht.

3 Weitere Potenziale liegen auch in der Verlagerung von Gütertransport im Lkw-Güterverkehr hin zum Schienengüterverkehr (Jahn et al., 2020) oder auch in der Vermeidung von Leerkilometerfahrten (für 2018 rund 6,6 Mrd. km; BMVI, 2020). Bedacht werden sollte hingegen, dass ein nicht unerheblicher Teil der Fahrleistung in Mio. km (nach Zulassungsstaaten) insbesondere von den Ländern Polen, Tschechien, Rumänien oder auch Litauen geleistet wird (BAG, 2020).

Abbildung 2
**Vergleich potenzieller Wasserstoffnutzung im Verkehr, 2019**

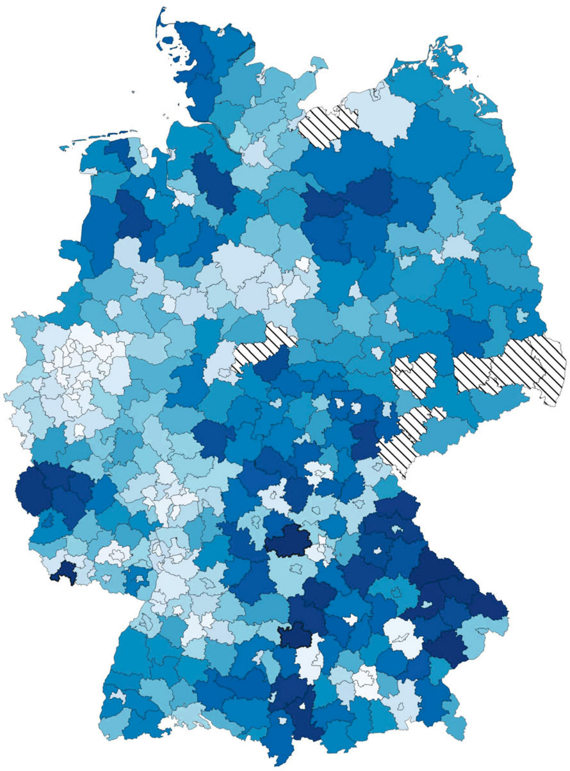

Hinweis: Die Farbskala ist nach Intensität abgestuft, wobei jedem Kreis eine eigene Farbgebung zukommt; schraffiert: keine Daten.

Quelle: Statistisches Bundesamt (2020); eigene Darstellung.

## Künftige Wasserstoffregionen in Deutschland

Die vorangestellte Analyse zeigt eine geografische Diskrepanz zwischen denjenigen Regionen in Deutschland, die Wasserstoff erzeugen, und den Regionen, die ihn derzeitig anwenden. Während erstere sich besonders in den norddeutschen Bundesländern (Windkraft) sowie in dünn besiedelten Regionen in Süd- und (Nord-) Ostdeutschland (Photovoltaik) konzentrieren, befinden sich die Schwerpunkte hinsichtlich des Bedarfs eher im Süden und Westen des Landes (Merten et al., 2020). Die Stellung Norddeutschlands als vielversprechende Wasserstoffregion wird weiter verstärkt durch den absehbaren Import von Wasserstoff, für den die Häfen wichtige Einspeisepunkte darstellen. Durch das zusätzliche Vorhandensein natürlicher Kavernen, die zur Wasserstofflagerung genutzt werden können, besteht die Chance, Norddeutschland als Distributionsschwerpunkt zu etablieren. Auch die hier konzentrierten Logistikverkehre wären für eine Wasserstoffumrüstung prädestiniert (Kruse et al., 2020).

Dieses Potenzial greifen dabei auch Forderungen auf, ein norddeutsches Cluster im Bereich der Wasserstoff- und Brennstoffzellentechnologie aufzubauen, Pilotregion für Wasserstoff- und wasserstoffbasierte Speicher zu werden und eine Wasserstoffgrundinfrastruktur bis nach Skandinavien für Logistikverkehre zu errichten. In diesem Zusammenhang sollen bis zum Jahr 2025 500 MW und bis 2030 5 GW Elektrolysekapazität in Norddeutschland aufgebaut werden (IHK Nord, 2019).

Entsprechend verweist auch Bayern in seiner Wasserstoffstrategie darauf, dass die benötigten Mengen an erneuerbarer Energie zur Wasserstofferzeugung nicht in Bayern allein erzeugt werden können und entsprechende Transportinfrastrukturen zu Offshore-Windparks oder großflächigen Photovoltaikanlagen in dünn besiedelten Gebieten nötig werden (STMWI, 2020). Gleichzeitig positioniert sich Nordrhein-Westfalen mit Verweis auf den hohen Industrieanteil und rechnet vor, dass rund 40 % des Wasserstoffbedarfs in Deutschland auf den Standort entfallen könnten (IN4Climate.NRW, 2019). Es wird somit deutlich, dass nicht nur auf sektoraler Ebene eine Priorisierung zugunsten jener Bereiche notwendig sein könnte, die für Wasserstoff besonders geeignet sein könnten, sondern auch auf regionaler Ebene, da die Produktionskapazitäten nicht zur Deckung des gesamten prognostizierten Wasserstoffbedarfs in Deutschland ausreichen werden.

## Handlungsempfehlungen

Um die geografische Differenz zwischen Erzeugungs- und Anwendungsregionen zu überwinden, ist der Aufbau von Lager- und Transportinfrastrukturen für Wasserstoff unumgänglich, wobei teilweise auf das vorhandene Erdgasnetz zurückgegriffen werden könnte. Weiterhin sind Wasserstofftankstellen notwendig, um auch eine kleinteilige Distribution im Verkehrsbereich darstellen zu können und Anreize für die strukturelle Transformation zu setzen (BMWi, 2020).

Hinzu kommen notwendige Impulse zur Nachfragesteigerung, um entsprechende Produktionsanreize und Sicherheiten für die Industrie zu gewährleisten (Hebling et al., 2019; Europäische Kommission, 2020b; IEA, 2019). Diskutiert werden außerdem Instrumente wie eine Quote für $CO_2$-frei erzeugten Wasserstoff, beispielsweise in Form von handelbaren Zertifikaten (Nymoen et al., 2019). In den Bereich staatlicher Handhabe über die Setzung von Anreizen hinaus ist eine Anpassung der regulatorischen Rahmenbedingungen für ein Hochlaufen der Wasserstofferzeugung in Deutschland notwendig. Aktuell macht die hohe Abgaben- und Steuerlast auf Strom den Betrieb von Elektrolyseuren zur Wasserstofferzeugung betriebswirtschaftlich unrentabel.

Notwendig könnte zudem eine Kostensenkung der Wasserstofferzeugung auf unter 500 Euro/kW sein. Forschungspotenziale bestehen hier vor allem in der Automatisierung der Produktion, die aktuell noch auf Manufakturniveau erfolgt (Hebling et al., 2019). Da bisher keine Massenfertigung von Elektrolyseuren etabliert worden ist, besteht für Unternehmen des Anlagen- und Maschinenbaus die Möglichkeit, hier zukünftig eine zentrale Position einzunehmen. Beim Aufbau einer Elektrolyseindustrie, die auch internationale Märkte bedienen kann, sind Maschinenbauer aus Deutschland mit ihren Kompetenzen und Wertschöpfungsketten in einer erfolgversprechenden Position (Hebling et al., 2019). Allein für deutsche Hersteller von Elektrolyseuren und Brennstoffzellen wird die mögliche Wertschöpfung auf 10 Mrd. Euro für 2030 bzw. 32 Mrd. Euro für 2050 taxiert (Hebling et al., 2019). Entlang der gesamten Wertschöpfungskette grünen Wasserstoffs leitet sich bis 2050 eine jährliche Bruttowertschöpfung von 2 Mrd. bis 30 Mrd. Euro, bzw. 20.000 bis 80.000 Arbeitsplätzen ab (Merten et al., 2020). Das Potenzial von grünem Wasserstoff ist immens. Es wurde bereits ein langer Weg der Entwicklung durchschritten, nun bedarf es einer zügigen und kurzfristigen Umsetzung, um im internationalen Wettbewerb den Trend in dieser technologischen und strukturellen Transformation zu setzen.
